# Nontuberculous mycobacteria skin infections associated with tattoos

**DOI:** 10.3389/fpubh.2026.1854792

**Published:** 2026-07-08

**Authors:** Soumana Daddy-Gaoh, Steven L. Foley, Ohgew Kweon, Seong-Jae Kim

**Affiliations:** Division of Microbiology, National Center for Toxicological Research, U.S. Food and Drug Administration, Jefferson, AR, United States

**Keywords:** microbial contamination, nontuberculous mycobacteria, skin infection, tattoo, tattoo ink

## Abstract

Nontuberculous mycobacteria (NTM) skin infections associated with tattoos and permanent makeup (PMU) have emerged as an important public health concern alongside the growing popularity of these procedures. Since the first reported case in 2003, increasing numbers have been documented worldwide. To better understand their epidemiology and clinical characteristics, we conducted a review of published cases. As of December 2024, 62 articles described 66 tattoo-related NTM skin infection cases, involving 310 individuals across 15 countries. Notably, 25 cases were outbreaks, accounting for 262 affected individuals, with the first in 2005 linked to contamination of a gray tattoo ink bottle. Ten NTM species were identified, most commonly *Mycobacterium chelonae* (32 cases), followed by *M. abscessus* (12 cases), and *M. haemophilum* and *M. fortuitum* (4 cases each). Contaminated tattoo ink, either diluted with nonsterile water during procedures or contaminated during manufacturing, was the primary source of infection; poor hygiene also contributed. Cutaneous symptoms typically appeared within weeks of tattooing, yet diagnostic delays were common due to nonspecific clinical presentations. Microbiological culture and molecular methods were the primary diagnostic tools. Treatment most often involved macrolide-based combination antibiotic therapy for extended periods, typically 3–6 months; surgical intervention was performed in some cases. Four cases were associated with PMU, emphasizing that infection risk extends beyond traditional tattooing. Collectively, these findings highlight the need for stricter infection control measures in tattoo practice, regulatory oversight of ink production, and increased clinical awareness to promote early diagnosis and effective management of tattoo-related NTM infections.

## 1 Introduction

Nontuberculous mycobacteria (NTM) comprise all mycobacterial species excluding the *Mycobacterium tuberculosis* complex and *M. leprae* (1, 2). These environmental microorganisms are widely distributed in natural and human-made environments, including soil, natural and treated water (1, 2). In humans, NTM can cause a range of infections, typically acquired from environmental sources rather than through person-to-person transmission (3, 4). In recent years, the clinical and public health significance of NTM infections has increased due to their rising incidence, growing antibiotic resistance, and diagnostic challenges (3, 4). Although pulmonary disease is the most common manifestation, NTM are also responsible for extrapulmonary infections, including lymphadenitis, skin and soft tissue infections, bone infections, and disseminated disease (4–6). Among these, cutaneous infections are the most frequently reported extrapulmonary manifestation, often associated with trauma, surgery, cosmetic procedures, or exposure to contaminated water (3–5, 7). These infections typically present as persistent, gradually worsening skin lesions, often emerging weeks to months after exposure, and are frequently misdiagnosed due to their nonspecific clinical appearance (4, 7, 8).

In parallel with the global rise in tattoo popularity (9, 10), reports of adverse events related to tattooing, including microbial infections, have also increased (11–13). Although the precise incidence of tattoo-related complications is unknown, various inflammatory, hypersensitivity, neoplastic, and infectious complications have been reported (14–16). Microbial infections, particularly bacterial infections, represent a significant proportion of these complications (17–20). Studies have estimated that approximately 1–6% of tattooed individuals report infections (13, 21). A marked increase in reported cases has been observed since 2000, with over 215 cases of microbial tattoo-related infections documented between 2000 and 2023, compared to 105 cases reported over the preceding century (19).

Permanent makeup (PMU) is a type of tattooing designed for cosmetic or aesthetic enhancements, including micropigmentation or microblading for eyebrows, eyeliner, lip tinting, and scar camouflage. Because PMU relies on the identical mechanisms of injecting pigment into the skin, it shares the same foundational techniques as traditional tattooing. For the purposes of this review, the terms ‘tattoo’ and ‘tattooing’ broadly incorporate all such procedures, encompassing PMU, microblading, and micropigmentation (19). From a regulatory standpoint, the FDA classifies both traditional tattoo and PMU inks as cosmetics, which do not require pre-market approval. Consequently, public health safety relies heavily on post-market surveillance, highlighting the need to monitor microbial contamination in both product types (22).

Among bacterial pathogens, NTM species have gained attention as a unique cause of tattoo-related skin infections. Historically, tattoo-related bacterial infections primarily involved *Staphylococcus aureus*, *Streptococcus pyogenes*, and *Pseudomonas aeruginosa* (19), which are common skin pathogens (23, 24). In contrast, NTM species are environmental organisms rarely associated with human skin infections, except under specific conditions. The clinical importance of NTM in this setting is probably driven by their ubiquitous presence in the environment, specifically in water systems, which poses a direct contamination risk for tattoo inks, and by specific biological characteristics, namely their lipid-rich cell wall, allowing them to persist in harsh conditions (1, 2) where other common pathogens might be eliminated. Since the first reported case of tattoo-related NTM infection in 2003, when a 27-year-old woman in Israel developed erythematous nodules 3 months after tattooing (25), the number of reported cases has increased worldwide (19). Since 2010, NTM infections have accounted for more than one-third of tattoo-related microbial infections (19, 20). Notably, many of these infections occurred in the context of outbreaks, often affecting multiple individuals simultaneously.

An interesting feature of tattoo-related NTM infections is their association with contaminated tattoo inks. For instance, in 2011, an outbreak of *M. chelonae* infections in New York was linked to contaminated gray tattoo ink, resulting in 19 infections and a manufacturer recall (26). Another outbreak in 2012 involving *M. abscessus* and *M. chelonae* infections in Washington State affected 31 individuals and was similarly linked to contaminated tattoo inks (27). These cases highlight the significant public health implications of NTM infections associated with tattoos and probably the unique epidemiological features that distinguish them from other tattoo-related microbial infections.

Despite the increasing number of reported cases and outbreaks, the epidemiology and clinical characteristics of tattoo-related NTM infections remain poorly understood. Therefore, in this systematic review, we analyzed 67 reported cases of tattoo-related NTM skin infections documented between 2003 and 2024. We examined the NTM species involved, sources of infection, outbreak patterns, clinical features, diagnostic approaches, treatment strategies, and recovery duration. This review aims to provide a comprehensive understanding of tattoo-related NTM infections and to inform future prevention and management strategies.

## 2 Case selection

A comprehensive literature search was conducted to identify all reports on NTM infections linked to tattooing. Permanent makeup (PMU) was also included in the search. PMU is a cosmetic form of tattooing, distinct from traditional tattoos, that uses similar techniques and is commonly applied for microblading and micropigmentation of the eyebrows, eyeliner, and lips (28, 29). In this article, tattooing refers to both traditional tattoos and PMU, with PMU mentioned separately when necessary. We searched Medline and Google Scholar for reports with no language restrictions. The initial search terms included “tattoo,” “permanent makeup,” and “nontuberculous mycobacteria.” To ensure a broad and inclusive search, the reference lists of included publications were manually reviewed and, additionally, we extended our search by analyzing articles that referenced previous studies on tattoo-related NTM infections. We identified case and outbreak reports, government health department reports, case review articles, conference presentation, and local or nationwide level survey reports, where details of cases are available. To ensure clarity, this article defines a “case” as an infectious event that takes place within the same period or geographic location, involving one or multiple patients, as long as it is linked to a common infection source, such as a specific tattoo ink, tattoo parlor, or tattooist. Therefore, an outbreak affecting multiple individuals is considered a single case under our definition.

A summary of NTM skin infections associated with tattoos is provided in Table 1 and Supplementary Table S1. The table outlines cases spanning from 2003 to 2024, detailing key epidemiological aspects, such as year of occurrence, NTM species identified, source of infection, ink type and color, number of individuals affected, outbreak status, time to symptom onset, time to diagnosis, antimicrobial treatments used, recovery duration, clinical characteristics, and geographic distribution.

**Table 1:** List of NTM infection cases related to tattoos.

Case #^a^	Year	# of individual (outbreak)^b^	NTM identified	Coinfected with^c^	Source of infection^d^	Type of ink	Tattoo color	Onset time^e^	Time to diagnosis (delayed)^f^	Antimicrobial treatment^g^	Recovery duration^h^	Clinical appearance^i^	Country	Reference
1	2003	1	*Mycobacterium* sp.	–	NA	Tattoo	NA	NA	3 mo (D)	NA	NA	Lesion/erythematous nodules	Israel	(25)
2	2003	1	*M. chelonae*	–	NA	Tattoo	NA	NA	NA	Ctx	NA	Nodule	Thailand	(56)
3	2005	20 (O)	*M. chelonae*	–	Contaminated ink (gray ink)	Tattoo	Gy	7–10 d	1–2 mo (D)	Clr/Tob	2 mo	Erythematous/inflammatory papulo-pustules/lesions	France	(30)
4	2005	8 (O)	*Mycobacterium* sp.	–	Contaminated ink (tap water)	Tattoo	Blk	2 wk	2–5 mo (D)	Min/Clr/Bac/Neo	1–2 mo	Erythematous papules/pustules/ulcerated nodules	France	(58)
5	2007	1	*Mycobacterium* sp.	–	NA	Tattoo	NA	NA	NA	NA	NA	NA	Ecuador	(59)
6	2007	48 (O)	*M. chelonae*	–	Contaminated ink (tap water)	Tattoo	Blk	3–35 d	2 mo	Clr/Tob	NA	Lesions/pustules	France	(31)
7	2007–2008	6 (O)	*M. chelonae*	–	Contaminated ink (tap water)	Tattoo	Gy	1–2 wk	4 mo (D)	Clr/Min/Azm	6 mo	Granulomatous papules/pustules/skin lesions	USA	(60)
8	2008	11 (O)	*M. abscessus*/*M. chelonae*	–	Tattoo ink (Dragon’g Blood Gray)	Tattoo	Gy	4–14 d	NA (D)	Dox	NA	Erythematous/papules	USA	(47)
9	2008–2012	2 (O)	*M. chelonae*	–	Contaminated ink (tap water)	Tattoo	Blk	NA	NA	Clr	NA	Erythema/postulation	UK	(61)
10	2008–2012	1	*M. chelonae*	–	NA	Tattoo	Gy	NA	NA	NA	NA	Erythema/postulation	UK	(61)
11	2008–2012	2	*M. chelonae*	–	NA	Tattoo	Gy	NA	NA	NA	NA	Erythema/postulation	UK	(61)
12	2008–2012	1	*M. chelonae*	–	NA	Tattoo	Gy	NA	NA	NA	NA	NA	UK	(61)
13	2008–2016	6 (O)	*Mycobacterium* sp.	–	NA	Tattoo	Gy	2 mo	NA	Cyclins	NA	Papules	Finland	(62)
14	2009	4 (O)	*M. chelonae*	–	Contaminated ink (industrial bolts)	Tattoo	Blk	3 wk	2 mo (D)	Clr/Mox	>4 mo	Erythema/edema/nodules	Australia	(63)
15	2009	12 (O)	*M. haemophilum*	–	Company’s makeup ink	PMU	NA	3 wk	1–3 mo (D)	Clr/Mox/Rif	7–9 mo	Inflammatory lesion/papules/pustules/plaque/abscess/fistulae	Switzerland	(39, 64)
16	2009	2 (O)	*M. haemophilum*	–	Contaminated ink (tap water)	Tattoo	Blk	3 d	2 mo (D)	Clr/Cip/Rif	6 mo	Erythematous nodules/pastulo-nodules	USA	(65)
17	2010	1	*M. abscessus*	–	Contaminated ink (tap water)	Tattoo	Gy	10 d	4 mo (D)	Clr/Min	4 mo	Erythematous papules/papulopustules/plaques	France	(66)
18	2010	2 (O)	*M. abscessus*	–	Contaminated ink (tap water)	Tattoo	Gy	1 wk	NA (D)	Clr/Min	5 mo	Papular eruption/erythematous non-pruritic papules	Australia	(67)
19	2010–2011	8 (O)	*M. fortuitum*	*S. aureus*	Tattooing/contaminated ink	Tattoo	Blk/Gy	3 d	>6 wk. (D)	Cip/Dox/Lzd	4–6 mo	Papules/plaques	USA	(68)
20	2010	7 (O)	*M. chelonae*	–	Tattooing/contaminated ink (water)	Tattoo	Gy	3–30 d	NA (D)	Clr	3–5 mo	Lesions/papules/superficial hyperkeratosis	Spain	(69)
21	2011	1	*M. chelonae*	–	NA	Tattoo	Gy	6 wk	2 wk. (D)	Clr/Lvx	6 mo	Erythematous plaques/pustules	USA	(70)
22	2011	1	*M. haemophilum*	–	Contaminated ink	PMU	NA	2 mo	NA (D)	Dox/Clr/Cip/Rif	6 mo	Cutaneous nodules/cysts	Germany	(57)
23	2011	7 (O)	*Mycobacterium* sp.	–	A Chinese company ink	PMU	Brn	1–2 wk	NA	Clr/Rif	3–6 mo	Granulomatous/purulent skin	Germany	(44)
24	2011	1	*M. immunogenum*	–	NA	Tattoo	NA	3 mo	NA (D)	Clr	9–12 mo	Lesions/erythematous papules/nodules	USA	(71)
25	2011	2 (O)	*M. chelonae*	–	Contaminated ink (tap water)	Tattoo	Blk	2–3 wk	NA (D)	Clr	NA	Pruritic/lichenoid papules/plaques with scales	Australia	(72)
26	2011	19 (O)	*M. chelonae*	–	Company A premixed gray ink	Tattoo	Gy	3 wk	NA (D)	Clr/Dox/Azm	NA	Erythematous rash	USA	(26)
27	2011	1	*Mycobacterium* sp.	–	Contaminated ink (tap water)	PMU	Tau	6 mo	5 mo (D)	Clr	4 mo	Erythematous plaques	USA	(40)
28	2011–2012	27 (O)	*M. abscessus*	–	Brand A black ink	Tattoo	Blk	1–3 wk	NA (D)	Mox/Clr/Cip/Lzd/Tig	2 mo	Papules/pustules/pruritus	USA	(27)
29	2011–2012	4 (O)	*M. chelonae*	–	Brand B graywash ink	Tattoo	Gy	1–3 wk	NA (D)	Mox/Clr/Cip/Lzd/Tig	4 mo	Papules/pustules/pruritus	USA	(27)
30	2012	2 (O)	*M. chelonae*	–	Company C ink	Tattoo	Blk	1 wk	NA	NA	NA	NA	USA	(73)
31	2012	1	*M. chelonae*	–	Contaminated ink (distilled water)	Tattoo	Blk	1 wk	NA	NA	NA	NA	USA	(73)
32	2012	2 (O)	*M. chelonae*	–	Contaminated ink (pharmacy water)	Tattoo	Gy	5–15 d	3–5 mo (D)	Clr	1–3 mo	Erythematous plaques/papulopustules	Spain	(74)
33	2012	1	*M. chelonae*	–	NA	Tattoo	NA	NA	NA (D)	Clr/Lvx,	9 mo	Erythema/nodular rash	USA	(75)
34	2012	1	*M. chelonae*	–	NA	Tattoo	Blk/Gy	2 wk	7 wk. (D)	Lzd/Azm	3 mo	Papules	USA	(76)
35	2012	1	*M. chelonae*	–	Contaminated ink	Tattoo	Gy	3 wk	2 wk	Clr	4 mo	Erythematous papule/pustules	USA	(77)
36	2012–2020	3	*M. chelonae*	–	NA	Tattoo	NA	NA	NA	NA	NA	NA	France	(78)
37	2013	1	*M. fortuitum*	–	NA	Tattoo	Blk	3 d	6 wk. (D)	Clr/Cip	10 mo	Cutaneous lesions/erythematous papules	Thailand	(79)
38	2013	4 (O)	*M. chelonae*	–	Contaminated gray ink	Tattoo	Gr	2 wk	3 wk. (D)	Clr	6 mo	Erythematous papules/pustules	UK	(80)
39	2014	1	*M. fortuitum*	–	NA	Tattoo	Blk	1–2 wk	NA (D)	Tmp/Cip	2 mo	non-pruritic papules	USA	(81)
40	2015	1	*M. abscessus*	–	NA	Tattoo	Gn	15 mo	NA (D)	Clr	5 mo	Lesion/erythematous papule-nodules	Brazil	(82)
41	2015	2	*M. mucogenicum*	–	NA	Tattoo	Blk	2 wk	NA	Clr/Amk/Cfx	5 wk	Pruritic erythematosquamous papules/plaques	Australia	(83)
42	2015	1	*M. fortuitum*	–	NA	Tattoo	Blk	2 wk	NA	Clr/Amk/Cfx	1 mo	Pruritic erythematosquamous papules/plaques	Australia	(83)
43	2015	1	*M. chelonae*	–	NA	Tattoo	NA	6 wk	NA	NA	NA	Erythematous papules/plaques/plaques	USA	(84)
44	2015	1	*M. chelonae*	–	NA	Tattoo	Red	2 wk	NA (D)	Clr/Dox	4 mo	Swelling keloid	USA	(85)
45	2015	38 (O)	*M. abscessus*	–	Contaminated ink (tap water)	Tattoo	Gy	1 wk	NA	Clr/Min	3–5 mo	Papules/pustules	USA	(33)
46	2016	6 (O)	*M. chelonae*/*M. abscessus*	–	NA	Tattoo	Gy	1 wk	NA (D)	Clr/Min	>2 mo	Erythematous pruritic papules/pustules	USA	(86)
47	2016–2021	2	*Mycobacterium* sp.	–	NA	Tattoo	NA	NA	NA	Dox	NA	NA	Finland	(87)
48	2017	1	*M. chelonae*	–	NA	Tattoo	NA	1 mo	NA	Clr	5 mo	pruritic papules	Germany	(88)
49	2017	1	*M. franklinii*	–	Tattooing	Tattoo	Gy	1 mo	2 mo (D)	Clr/Cip	NA	Papules	USA	(89)
50	2017	11 (O)	*M. abscessus*	–	Tattooing	Tattoo	Blk/Gn	2–8 wk	10 d (D)	Clr	6 wk	Erythematous papules	Taiwan	(90)
51	2017–2021	1	*Mycobacterium* sp.	–	Tattooing	Tattoo	NA	NA	NA	NA	NA	NA	Netherlands	(91)
52	2018	1	*M. haemophilum*	–	NA	PMU	NA	1 wk	1 mo (D)	Clr/Cip/Rif	2 mo	Pruritic papulonodular/erythematous lesions	Thailand	(92)
53	2018	1	*M. massiliense*	–	Tattooing	Tattoo	Gy	2 wk	1 mo (D)	Azm	3 mo	Erythematous papular rash/lesions	USA	(93)
54	2020	1	*M. mageritense*	–	NA	Tattoo	Gy	3 wk	1 mo	Min/Mox	3 mo	Erythematous papules/pustules/plaques	USA	(43)
55	2020	1	*M. abscessus*	–	NA	Tattoo	Gy	10 d	NA (D)	Clr/Mox	4 mo	Erythematous papules	Australia	(94)
56	2020	1	*M. franklinii*	–	NA	Tattoo	Blu	NA	NA (D)	Clr/Dox	9 mo	Papules	USA	(95)
57	2020	1	*M. kansasii*	–	NA	Tattoo	NA	3–5 yr	3 mo	Inh/Rbt/Azm/Pza/Emb	5 mo	Skin lesions/papules/nodules/verrucous plaques	USA	(96)
58	2020	4 (O)	*M. chelonae*	–	Contaminated ink (tap water)	Tattoo	Blk	NA	NA	Clr/Azm/Lvx	4 mo	Erythematous papules/pustules	UK	(32)
59	2020	1	*Mycobacterium* sp.	–	Contaminated ink	Tattoo	Gy	NA	NA	Clr/Dox/Mox	NA	Erythematous papules/plaques	USA	(97)
60	2021	1	*M. mageritense*	–	Contaminated ink (nonsterile water)	Tattoo	Blk	1 mo	5 wk. (D)	Cip/Tmp/Smx	5 wk	Erythematous papules/pustules	Australia	(98)
61	2021	1	*M. abscessus*	–	Contaminated ink (nonsterile water)	Tattoo	Blk/Gy	2 wk	NA	Clr/Amk/Cfx/Ipm	6 wk	Erythematosquamous papules/pustules	Netherlands	(45)
62	2022	1	*M. abscessus*	*Nocardia* sp.	NA	Tattoo	Blk/Gy	2 wk	6 wk. (D)	Ipm/Amk/Omd/Emb/Azm	9 mo	Erythematous papules/pustules	USA	(41)
63	2022	3	*Mycobacterium* sp.	–	NA	Tattoo	NA	NA	NA	NA	NA	NA	USA	(99)
64	2022	1	*M. abscessus*/*M. chelonae*	–	NA	Tattoo	Blk	NA	NA (D)	Cip/Azm	6 mo	Erythematous nodules/pustules	Thailand	(100)
65	2023	1	*M. chelonae*	–	NA	Tattoo	NA	2 wk	2 wk. (D)	Azm/Lzd	6 mo	Erythematous papules/pustules	Germany	(101)
66	2023	1	*M. massiliense*	–	NA	Tattoo	NA	3 d	NA	Dox/Min/Ctx/Rif	3 mo	Nodular lesion	Mexico	(102)
67	2024	1	*M. chelonae*	–	Tattooing	Tattoo	Blk	1 wk	NA	Clr	NA	Erythema/pruritus/Hyperkeratotic/hyperpigmented plaques	USA	(103, 104)

a A case is defined as an infectious event occurring within the same timeframe or geographic area, whether it involves one person or multiple individuals. This event is usually, if not always, linked to a common source of infection, such as a specific brand of tattoo ink, a tattoo parlor, or a tattoo artist. Therefore, under our definition, an outbreak involving multiple individuals is classified as a single case.

b “O” indicates an outbreak. Some cases involving multiple individuals are not classified as outbreaks when it is unclear whether they originate from a common source of infection.

c Abbreviation for bacterial genus name: *S*., *Staphylococcus.*

d “Tattooing” as a source of infection typically involves insufficient hygienic practices or conditions during the tattoo procedure, such as the use of contaminated tattoo instruments.

e Abbreviations for time periods: d, day; wk., week; mo, month; yr., year.

f “D” indicates a delayed diagnosis. Cases are considered delayed diagnosis when the infection is referred to a secondary provider or the initial treatment fails.

g Abbreviations for antibiotics: Ctx, cotrimoxazole; Clr, clarithromycin; Tob, tobramycin; Min, minocycline; Bac, bacitracin; Neo, Neomycin; Azm, azithromycin; Dox, doxycycline; Cyc, cyclins; Mox, moxifloxacin; Rif, rifampin; Cip, ciprofloxacin; Lzd, linezolid; Lvx, levofloxacin; Tgc, tigecycline; Tmp, trimethoprim; Amk, amikacin; Cfx, cefoxitin; Iso, isoniazid; Rbt, rifabutin; Azm, azithromycin; Pza, pyrazinamide; Emb, ethambutol; Smx, Sulfamethoxazole; Ipm, imipenem; Omd, omadacycline; Ctx, ceftriaxone.

h It indicates the time required for recovery, typically measured from the point of diagnosis and the start of antimicrobial treatment.

i Clinical appearance as described in the respective case report.

## 3 Epidemiology of tattoo-related NTM infections

### 3.1 Incidence and outbreak trends

Since the first case in 2003 up to 2024, we have identified 63 articles documenting 67 cases of NTM skin infection after receiving tattoos, affecting 311 individuals (Table 1). Usually, 2–5 infection cases occurred each year, except in 2011 and 2012, when there were 9 and 7 cases, respectively. Infection cases have been reported in 15 countries worldwide, with the USA having the highest number at 29 cases, followed by the UK and Australia with 6 cases each, France with 5 cases, and Germany and Thailand with 4 cases each.

Twenty-five outbreak cases, accounting for over one-third of all cases, were identified (Table 2). The earliest recorded outbreak, in 2005 in France, involved 20 individuals infected with *M. chelonae* due to contamination of a bottle of gray ink used by a single tattoo artist (30). The largest outbreak occurred in 2007, affecting 48 individuals with *M. chelonae*, where tap water used to dilute tattoo ink was identified as the infection source (31). The most recent outbreak, in 2020, involved 4 individuals infected by *M. chelonae*, with the source traced to tap water used for diluting black ink (32). In the US, 10 outbreaks have been reported, including the two cases in New York and Washington State mentioned in the introduction, as well as the second-largest outbreak involving *M. abscessus* (33). This outbreak occurred in Florida in 2015, affecting 38 individuals who developed infections after receiving tattoos at a single studio. The infections were linked to tattoo inks contaminated during manufacturing.

**Table 2:** Summary of clinical and microbiological data of the tattoo-related NTM skin infections.

Variable	Total	Rapid growing NTM								Slow growing NTM		NTM species unidentified
		*M. chelonae*	*M. abscessus*	*M. fortuitum*	*M. franklinii*	*M. immunogenum*	*M. mageritense*	*M. massiliense*	*M. mucogenicum*	*M. haemophilum*	*M. kansasii*	
# of case	67	32	12	4	2	1	2	2	1	4	1	10
# of infected individual	311	160	69	12	2	1	2	2	2	16	1	32
# of outbreak	25	15	6	1	–	–	–	–	–	2	–	3
# of infected individual from outbreak	262	141	95	8	–	–	–	–	–	14	–	21
Time to symptom onset (case)												
≤1 wk	17	8	4	2	–	–	–	1	–	2	–	–
1–4 wk	33	16	7	2	1	–	2	1	1	1	–	2
4–8 wk	6	3	1	–	–	–	–	–	–	1	–	1
>8 wk	4	–	1	–	–	1	–	–	–	–	1	1
Time to diagnosis (case)												
≤1 mo	8	4	1	–	–	–	1	1	–	1	–	–
1–3 mo	14	4	1	2	1	–	1	–	–	2	1	2
>3 mo	5	2	1	–	–	–	–	–	–	–	–	2
Cases with missed initial diagnosis	45	17	11	3	2	1	2	1	–	4	–	4
Cases referred to secondary care	23	9	4	1	2	1	–	–	–	4	–	2
Cases initially treated with steroids	33	15	9	2	2	1	2	–	–	–	–	2
Cases initially treated with inappropriate antibiotics	25	9	7	1	–	1	–	1	–	3	–	3
Method of NTM identification (case)												
Culture	54	28	12	4	2	1	2	1	1	3	–	–
HPLC or mass spectrometry	2	1	–	–	–	–	1	–	–	–	–	–
Molecular method	20	5	6	–	1	1	–	–	–	4	1	2
AFB positive	22	6	2	3	1	1	1	–	–	2	1	5
Cases with frequently used antibiotics												
Clarithromycin	42	19	9	2	2	1	–	–	1	4	–	4
Azithromycin	10	6	2	–	–	–	–	1	–	–	1	–
Doxycycline	10	3	1	1	1	–	–	1	–	1	–	2
Minocycline	9	2	4	–	–	–	1	1	–	–	–	1
Ciprofloxacin	12	2	2	3	1	–	1	–	–	3	–	–
Moxifloxacin	7	2	2	–	–	–	1	–	–	1	–	1
Levofloxacin	3	3	–	–	–	–	–	–	–	–	–	–
Rifampin	6	–	–	–	–	–	–	1	–	4	–	1
Linezolid	5	3	1	1	–	–	–	–	–	–	–	–
Amikacin	4	–	2	1	–	–	–	–	1	–	–	–
Cases treated with a single antibiotic	17	10	3	–	–	1	–	1	–	–	–	2
Cases treated with combination therapy	40	14	9	4	2	–	2	1	1	4	1	4
Time to recovery (case)												
≤1 mo	1	–	–	1	–	–	–	–	–	–	–	–
1–3 mo	16	4	4	1	–	–	2	2	1	1	–	1
3–6 mo	24	12	6	1	–	–	–	–	–	2	1	2
>6 mo	6	1	1	1	1	1	–	–	–	1	–	–

While pulmonary or extrapulmonary NTM infections in most cases occurred as isolated cases, often affecting only a single individual, a substantial proportion of NTM skin infections presented as outbreaks involving multiple individuals, making this a notable epidemiological characteristic of tattoo-associated infections. As shown in Table 2, these outbreaks affected 262 individuals, accounting for over 80% of the 311 total individuals. This suggests that although outbreaks are relatively less common, they have a significant impact, affecting a large number of individuals when they do occur.

### 3.2 NTM species identified in tattoo-related infections

We identified at least 10 distinct NTM species associated with tattoo-related skin infections (Table 2). *M. chelonae* was the most frequently reported, accounting for nearly half of the cases (32 cases), followed by *M. abscessus* (12 cases) and *M. fortuitum* (4 cases), all of which belong to the rapidly growing mycobacteria (RGM) group (34, 35). These species are among the most commonly reported NTMs in skin and soft tissue infections (3, 4, 36). Other RGM species identified in a few cases included *M. franklinii*, *M. immunogenum*, *M. mageritense*, *M. massiliense*, and *M. mucogenicum*, among which *M. franklinii* and *M. immunogenum* had not previously been reported in human skin infections. Additionally, two slowly growing mycobacteria (SGM), *M. haemophilum* and *M. kansasii*, were also identified. More prevalent NTM species commonly associated with skin infections, such as *M. avium*, *M. marinum*, and *M. ulcerans* (4, 36, 37), were not observed in tattoo-associated cases. This absence may be attributed to differences in transmission routes, environmental sources, or ecological niches and is considered a characteristic feature of the specific NTM species implicated in tattoo-related infections.

## 4 Clinical features and diagnostic challenges

### 4.1 Symptom onset and clinical manifestations

The onset of symptoms in tattoo-related NTM infections showed substantial variability (Table 1). While most cases developed symptoms within 1 to 4 weeks after tattoo placement, onset ranged from as early as 3 days to over a year. In particular, 17 cases presented within the first week, and 33 cases between 1 and 4 weeks. A delayed onset of more than 8 weeks was noted in 4 cases, including one *M. kansasii* infection that appeared 3 to 5 years after tattooing. Infections caused by RGM, such as *M. chelonae* and *M. abscessus*, were typically associated with symptom onset within a few weeks.

**Figure 1:**
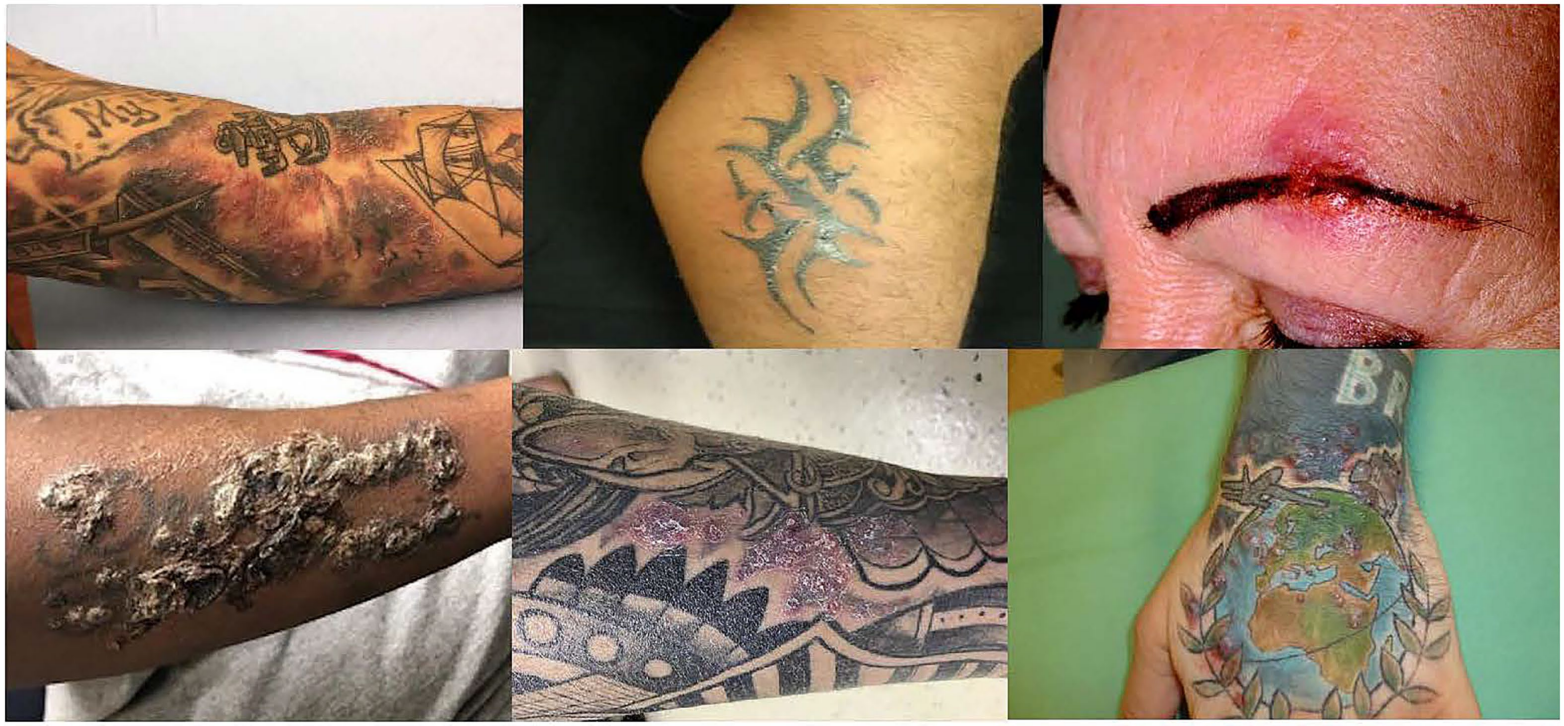
Examples of NTM skin infections associated with tattoo or permanent makeup. **(A)** Erythematous plaques studded with pustules located predominantly in the shaded portions of ink, caused by *M. mageritense* infection. Reproduced with permission from Dermatology Online Journal via the original article by Park et al., 2020, 26. **(B)** Nodular lesion on the right leg under a tattoo, caused by *M. massiliense* infection. Reproduced from Rodríguez-Cerdeira et al., 2022, Pathogens, 11, licensed under CC BY 4.0. **(C)** An erythematous plaque with ulceration on the left eyebrow of a patient following PMU, caused by *M. haemophilum* infection. Reproduced with permission from the journal and author; Hamsch et al., 2011, Acta Dermato-Venereologica, 89. **(D)** Verrucous plaques overlying long-standing tattoos caused by *M. kansasii* infection. Reproduced from Gupta et al., 2020, SKIN The Journal of Cutaneous Medicine, 4, licensed under CC BY 4.0. **(E)** Scaling erythematous papules and pustules coalescing into plaques on the left lower leg resulting from tattoo-inoculated *M. mageritense* infection. Reproduced from Lobo et al., 2021, Case Reports in Dermatology, 13, licensed under CC BY-NC 4.0. **(F)** Multiple erythematous papules and pustules up to 3 mm in diameter within the tattooed area on the dorsal hand, caused by *M. chelonae* infection. Reproduced from Lange et al., 2023, Dermatologie (Heidelb), 74, licensed under CC BY 4.0.

The clinical presentation of tattoo-related NTM infections varies widely, but most cases involve chronic, slowly progressing lesions that are often misdiagnosed due to their nonspecific appearance (Table 1 and Figure 1). The most frequently reported clinical manifestations include erythematous papules, pustules, and nodules, with some cases progressing to plaques, cysts, or ulcerations. Granulomatous reactions were observed in certain infections, while other presentations included pruritus, scaling, and edema.

The histological appearance of tattoo-related NTM infections primarily shows granulomatous inflammation, characterized by epithelioid histiocytes, multinucleated giant cells, and lymphocytic infiltration (Supplementary Table S1). In several cases, suppurative granulomas with central necrosis were observed. Some infections exhibited mixed inflammatory cell infiltration, including neutrophils and plasma cells, suggesting an ongoing immune response. Acid-fast bacilli (AFB) were detected on Ziehl-Neelsen staining in about one-third of cases (22 out of 67).

### 4.2 Diagnosis

The time to diagnosis of tattoo-related NTM infections varied widely, ranging from 10 days to several months (Table 2). While 8 cases were diagnosed within one month, the majority required a prolonged diagnostic period of 1 to 3 months (14 cases), and in 5 cases, confirmation took more than 3 months. These extended diagnostic timelines were often attributed to initial misdiagnoses, which remain a persistent challenge in dermatology and infectious disease management. As summarized in Table 2, 45 cases (approximately 70%) were either misdiagnosed or initially undiagnosed, with 23 cases requiring referral to secondary clinics due to diagnostic uncertainty or incorrect initial diagnoses by primary healthcare providers. Notably, 33 cases, representing nearly half of all reported cases, were initially treated with corticosteroids, likely due to misinterpretation as inflammatory or allergic skin conditions. Furthermore, 25 cases received inappropriate antibiotic therapy ineffective against NTM, contributing to further delays in establishing the correct diagnosis and initiating effective treatment. Notably, all four cases of *M. haemophilum* infection required referral to secondary clinics, and in three of these cases, patients were initially treated with inappropriate antibiotics.

The high rate of initial misdiagnosis in tattoo-related NTM infections arises from several key factors. First, the nonspecific clinical presentation, including papules, nodules, and lesions, often mimics bacterial or fungal infections, inflammatory skin conditions, or allergic reactions (3). This frequently leads to incorrect empirical treatments, particularly corticosteroids and often inappropriate or broad-spectrum antibiotics, which are ineffective against NTM and may worsen disease progression. Second, microbiological challenges contribute to diagnostic delays (4). NTM grow slowly, often requiring weeks to over a month for detection, in contrast to faster-growing microbes. The prolonged incubation time required for NTM tissue culture can lead to false-negative AFB staining results due to low bacterial load (3, 38). Among 43 cases in which AFB staining was attempted (Supplementary Table S1), we observed that half (21 cases) yielded negative results, indicating that a negative AFB stain does not rule out NTM infection. Additionally, certain NTM species require specific growth conditions for successful isolation. For example, *M. haemophilum* requires iron supplementation and a lower incubation temperature, making its detection challenging using standard laboratory protocols (4). In one reported case, it took 70 days to culture *M. haemophilum* and achieve species-level identification (39). As a result of these demanding culture conditions, cases of tattoo-related *M. haemophilum* infection are suspected to be underrecognized. Furthermore, harsh decontamination procedures, often used to eliminate contaminants in culture media, may also reduce NTM viability, further complicating successful culture recovery (4). Finally, co-infections with other bacterial species can further obscure diagnosis, as overlapping clinical manifestations complicate NTM identification. Three such cases were documented (Table 1). In a 2011 case report (40), a patient developed a persistent skin infection following a cosmetic tattoo, where NTM was initially undetected and later found alongside methicillin-resistant *S. aureus*. It took 5 months to detect the NTM, leading to delayed diagnosis and prolonged ineffective treatment before appropriate antimycobacterial therapy was initiated. A 2022 case study described a lung transplant recipient who developed a severe *M. abscessus* skin infection following tattooing, which was initially misdiagnosed as a *Nocardia* infection, leading to ineffective initial treatment (41).

### 4.3 NTM identification methods

NTM species identification was primarily performed using culture-based methods, which were applied in 54 cases, making it the most frequently used technique. Molecular methods, such as PCR-based assays, were utilized in 20 cases, including species like *M. haemophilum*, *M. franklinii*, and *M. kansasii*, where precise genetic identification was necessary. AFB staining yielded positive results in about half of the cases where it was performed, serving as an initial diagnostic tool for detecting mycobacterial infections. Additionally, high-performance liquid chromatography (HPLC) or mass spectrometry was used in 2 cases for species identification. Given the complexity of NTM identification, a combination of methods is often recommended to ensure accuracy (8). In our analysis, 35 cases employed more than two identification methods, primarily combining culture and molecular techniques, with 7 of these cases identified using three different methods.

## 5 Treatment strategies and recovery

### 5.1 Antimicrobial treatment approaches

The antibiotic treatment of tattoo-related NTM infections varied depending on the species involved, with clarithromycin being the most frequently used antibiotic, prescribed in 42 cases, particularly for *M. chelonae* and *M. abscessus* (Table 2). Another macrolide antibiotic, azithromycin, was used in 10 cases. Tetracycline antibiotics, including doxycycline (10 cases) and minocycline (9 cases), were commonly administered for other species. Fluoroquinolones such as ciprofloxacin (12 cases), moxifloxacin (7 cases), and levofloxacin (3 cases) were also used. Additional antibiotics, including rifampin (6 cases), linezolid (5 cases), and amikacin (4 cases), were administered in select cases.

Combination therapy is often recommended for the treatment of NTM infections, particularly for drug-resistant strains, to enhance treatment effectiveness (37). In our analysis, combination therapy was used in 40 cases, while 17 cases were treated with a single antibiotic. The most frequently used combination involved the macrolide clarithromycin and tetracycline antibiotics, administered in 17 cases (minocycline, 9 cases; doxycycline, 5 cases; tigecycline, 2 cases). Another common regimen was clarithromycin combined with fluoroquinolones, used in 14 cases, incorporating ciprofloxacin, moxifloxacin, or levofloxacin. Triple-combination therapy was employed in 19 cases, typically involving antibiotics from different classes. Notably, all four cases of *M. haemophilum* infection were managed with triple-combination therapy, which included a macrolide (clarithromycin), a fluoroquinolone (ciprofloxacin or moxifloxacin), and either a tetracycline (doxycycline) or a rifamycin (rifampin) antibiotic.

### 5.2 Clinical recovery and duration

The duration of recovery following antibiotic treatment varied widely. Among the 47 cases where this information was available, recovery occurred within 1 month in only one case, while 16 cases resolved within 1 to 3 months, suggesting relatively rapid responses. However, the majority of cases (24 cases) required 3 to 6 months to achieve resolution, which was the most common timeframe, observed not only in *M. chelonae* and *M. abscessus* infections but also in slower-growing species like *M. haemophilum* and *M. kansasii*. Prolonged recovery times exceeding 6 months were observed in 6 cases, involving *M. fortuitum*, *M. franklinii*, *M. haemophilum*, and *M. abscessus*. These observations align with the widely accepted, yet variable, recommendations for treatment duration. Although no standardized guidelines exist, and several factors, such as infection severity, treatment regimens, and antibiotic susceptibility, influence recovery times, antibiotic therapy for 3 to 12 months is commonly recommended (4, 42, 43). Overall, antibiotic combinations and treatment durations were highly diverse, based on species-specific resistance patterns, severity of infection, and each patient’s clinical response.

Spontaneous recovery from tattoo-related NTM infections without antibiotic treatment is probably very rare, but has been observed. One patient with a granulomatous skin lesion recovered without intervention after permanent makeup (44). A case of *M. abscessus* infection resolved within 6 weeks without antibiotics (45).

## 6 Sources of infection and risk factors

### 6.1 Source of NTM infection

Microbial infections linked to tattoos may originate from several sources, including the tattooist, if they are a carrier of pathogens, or from poor hygiene practices, such as the use of nonsterile instruments or sharing contaminated needles. Additionally, contamination can occur when tattoo inks are diluted with nonsterile water, such as tap water, during the tattooing process. Tattoo inks may also become contaminated during manufacturing, posing a risk of infection (15, 18, 46). Furthermore, infections can develop if proper aftercare is not followed during the healing period.

Our analysis revealed that the most common source of tattoo-associated NTM infections was contamination of tattoo inks during procedures, particularly through dilution with nonsterile water such as tap water. This accounted for 22 cases involving 127 individuals (Table 3 and Supplementary Table S1). Insufficient hygiene during tattooing was identified as the source in 7 cases involving 30 individuals. Additionally, tattoo ink contaminated during manufacturing was responsible for 8 cases involving 120 individuals. These findings indicate that tattoo ink contamination, whether during procedures or manufacturing, was the main source of infection in 30 out of 37 cases (81%) where the source was identified. Notably, when tattoo inks were the source of infection, they were frequently associated with outbreaks. Of the 30 cases linked to contaminated tattoo ink, 22 cases resulted in outbreaks. Given that the total number of NTM outbreaks was 25, it is evident that when tattoo inks are contaminated, there is a high likelihood of outbreak events affecting multiple individuals. Furthermore, in all 8 cases where ink contamination occurred during manufacturing, the infections led to outbreaks. In at least 2 of the 8 cases, the tattoo inks involved in the outbreaks were recalled by the manufacturer (26, 27).

**Table 3:** Cases of NTM infection based on source of infection.

Source of infection^a^	# of case^b^	# of infected individual^b^
Insufficient hygiene during tattooing	7 (3)	30 (26)
Contaminated ink in procedure	22 (14)	127 (119)
Manufacturer-contaminated ink	8 (8)	120 (120)

a In two cases, both insufficient hygiene and contaminated ink during tattooing were simultaneously identified as sources of infection. These cases were counted in both categories.

b Number in parentheses indicates those involved in outbreaks.

### 6.2 Outbreak dynamics and health relevance

Infection resulting from contaminated tattoo inks is a distinctive feature of tattoo-related NTM infections. Unlike other sources, these infections frequently lead to outbreaks affecting multiple individuals from a single contaminated source and often involve geographically unrelated locations, particularly when contamination occurs at the manufacturing level, thereby complicating epidemiological efforts to trace the origin of infection. For example, the first such case occurred in 2008 in San Antonio, affecting 11 individuals. A tattoo ink branded as “Dragon’s Blood Gray,” manufactured in New Jersey, was sent to a distributor in California before being mailed to the tattoo parlor in San Antonio (47). In the 2012 outbreak mentioned in the introduction, while 5 individuals were confirmed to be infected with NTM, the ink manufacturer received 35 complaints of skin reactions from 19 U.S. states during the same period (27). In another outbreak from 2011 to 2012, involving 19 confirmed cases and an additional 27 possible cases, a brand of ink from a manufacturer was identified as the source in two states, Washington and Iowa, simultaneously (26). These examples highlight the critical impact of manufacturing processes on tattoo ink safety and the consequent risk of infection. Although no such outbreak cases have been observed since 2012, over the years, multiple studies have consistently reported that a significant proportion of marketed tattoo inks, ranging from 10 to 86%, are contaminated with microorganisms, including potentially pathogenic species (22, 48–55). Data from the U.S. Food and Drug Administration and the European Union’s Safety Gate^1^ document instances of tattoo inks recalled from the market due to microbial contamination.

## 7 Other epidemiological considerations

### 7.1 Traditional tattooing vs. PMU

We identified five cases of NTM infection associated with PMU. Among these five cases, four were linked to ink contamination, with two of them attributed to manufacturer-level contamination. These findings suggest that, although PMU is generally regarded as safer than traditional tattooing, often performed in more controlled settings, the risk of NTM infection persists.

### 7.2 Professional vs. nonprofessional tattooing

Among the 40 cases where information about the location and the person performing the tattooing was available, four were carried out by nonprofessional tattooists. With the increasing availability of do-it-yourself tattoo kits on the market, it remains difficult to estimate how many individuals undergo tattooing in uncontrolled, nonprofessional environments. In any case, these cases demonstrate that tattoos, whether performed inprofessional, regulated settings or not, carry a risk of infection, emphasizing the importance of proper hygiene, safe practices, and product safety regardless of where the procedure is conducted.

### 7.3 Surgical intervention cases

Surgical intervention was infrequently reported and was performed in three documented cases of tattoo-related NTM skin infections. In one case reported in 2003, the patient underwent surgical excision in combination with antibiotic therapy, resulting in a favorable outcome without relapse (56). Wollina et al. described a case of *M. haemophilum* infection associated with permanent makeup, in which the patient received surgical excision of a nodule followed by antibiotic treatment, leading to complete clinical remission (57). Furthermore, in an outbreak of *M. haemophilum* infections reported in 2011, 10 out of 12 patients required surgical procedures, including partial parotidectomy, lymph node excision, or fistula resection, due to inadequate response to antimicrobial therapy or intolerance to prolonged treatment (39).

## 8 Conclusion

This systematic review provides a comprehensive overview of tattoo- and PMU-related NTM skin infections reported over the past two decades. Our analysis revealed that these infections pose significant public health concern due to their potential for outbreaks and prolonged clinical course. Contaminated tattoo ink, either introduced during manufacturing or diluted with nonsterile water during tattooing, was identified as the primary source of infection. Poor hygienic practices further contributed to infection risk. Notably, the majority of outbreak cases were linked to contaminated inks, highlighting the critical role of tattoo ink quality and handling in infection prevention.

The clinical features of tattoo-related NTM infections were diverse and often nonspecific, leading to diagnostic delays and frequent initial misdiagnoses. Culture and molecular methods were essential for accurate species identification, with *M. chelonae* and *M. abscessus* being the most commonly implicated species. Treatment strategies were diverse but most frequently involved macrolide-based combination antibiotic therapy over several months. These findings emphasize the need for heightened clinical awareness, adherence to strict infection control practices in tattooing procedures, and regulatory oversight of tattoo ink production and use. Future public health efforts should focus on improving surveillance, ensuring product safety, and developing standardized guidelines for the diagnosis and management of tattoo-related NTM infections.

## Footnotes

1
https://ec.europa.eu/safety-gate

## Supplementary material

SUPPLEMENTARY table S1 Published case reports of tattoo-related nontuberculous mycobacteria (NTM) infections.
